# MicroRNAs Can Influence Ovarian Cancer Progression by Dysregulating Integrin Activity

**DOI:** 10.3390/cancers15184449

**Published:** 2023-09-08

**Authors:** Zacharias Fasoulakis, Michaela-Zoi Psarommati, Angeliki Papapanagiotou, Vasilios Pergialiotis, Antonios Koutras, Athanasios Douligeris, Anastasia Mortaki, Antonios Mihail, Marianna Theodora, Sofoklis Stavros, Defkalion Karakalpakis, Maria Papamihail, Emmanuel N. Kontomanolis, George Daskalakis, Panos Antsaklis

**Affiliations:** 1 1st Department of Obstetrics and Gynecology, National and Kapodistrian University of Athens, 115 28 Athens, Greece; pergialiotis@yahoo.com (V.P.); antoniskoy@yahoo.gr (A.K.); thanosdouligeris92@gmail.com (A.D.); anastasiamort@gmail.com (A.M.); tonismiha@hotmail.gr (A.M.); martheodr@gmail.com (M.T.); dkarakalpakis@gmail.com (D.K.); mapapam@hotmail.com (M.P.); 2 Department of Obstetrics and Gynecology, Democritus University of Thrace, 681 00 Alexandroupolis, Greece; psaromika@gmail.com (M.-Z.P.); mek-2@otenet.gr (E.N.K.); 3 Laboratory of Chemistry Biology, National and Kapodistrian University of Athens, 115 28 Athens, Greece; 4 3rd Department of Obstetrics and Gynecology, National and Kapodistrian University of Athens, Medical School, Attikon Hospital, 124 62 Athens, Greece; sfstavrou@yahoo.com; 5 1st Department of Obstetrics and Gynecology, National and Kapodistrian University of Athens, 106 76 Athens, Greece; gdaskalakis@yahoo.com (G.D.); panosant@gmail.com (P.A.)

**Keywords:** miRNAs, integrins, ovarian cancer, therapeutic targets

## Abstract

**Simple Summary:**

MicroRNAs (miRNAs) are small RNA molecules that regulate gene expression, with significant implications in ovarian cancer development and progression. Their dysregulation can act as either oncogenes or tumor suppressors in ovarian cancer. For instance, the miR-200 family is associated with increased invasive capabilities in cancer cells, while the let-7 family controls cell proliferation and migration. Moreover, miRNAs have shown involvement in ovarian cancer chemoresistance, such as the role of miR-21 in cisplatin resistance. The potential of miRNAs as diagnostic and prognostic biomarkers is being investigated, with certain miRNAs correlating with tumor stage and survival. Therapeutic strategies, including miRNA mimics and inhibitors, are being developed to target dysregulated miRNAs, showing promise in preclinical studies. Another area of focus is the relation between miRNAs and integrins, proteins crucial for cell migration, adhesion, and proliferation. Specific miRNAs have been found to regulate ovarian cancer progression by targeting integrin mRNA, thereby influencing cancer cell activities. This relationship underscores the therapeutic potential of targeting miRNAs and integrins in ovarian cancer treatment, pointing to a novel direction for research and clinical application.

**Abstract:**

Ovarian cancer is a deadly disease that affects thousands of women worldwide. Integrins, transmembrane receptors that mediate cell adhesion and signaling, play important roles in ovarian cancer progression, metastasis, and drug resistance. Dysregulated expression of integrins is implicated in various cellular processes, such as cell migration, invasion, and proliferation. Emerging evidence suggests that microRNAs (miRNAs) can regulate integrin expression and function, thus affecting various physiological and pathological processes, including ovarian cancer. In this article, we review the current understanding of integrin-mediated cellular processes in ovarian cancer and the roles of miRNAs in regulating integrins. We also discuss the therapeutic potential of targeting miRNAs that regulate integrins for the treatment of ovarian cancer. Targeting miRNAs that regulate integrins or downstream signaling pathways of integrins may provide novel therapeutic strategies for inhibiting integrin-mediated ovarian cancer progression.

## 1. Introduction

### 1.1. Epithelial Ovarian Cancer

Ovarian carcinoma constitutes one of the deadliest gynecological cancers in the developed world, with epithelial ovarian carcinoma (EOC) being the most common type. The vast majority of women suffering from ovarian cancer present at cancer stages III–IV at the time of diagnosis, primarily due to their nonspecific symptomatology (abdominal bloating and pain, nausea, early satiety, and alteration in bowel habits) and the lack of a reliable screening test [1].

The spectrum of factors contributing to the pathogenesis of ovarian cancer is wide. Genetic predisposition has been proven to play a crucial role in the progression of ovarian cancer, principally through mutations of BRCA1–BRCA2 and mismatch repair (MMR) genes. Another correlation has been found between ovarian cancer and increased ovulatory cycles. Other parameters include endometriosis, dietary factors (a diet low in fiber and low in vitamin D), and ethnicity [2].

The World Health Organization (WHO) classifies EOC into five dominant histotypes: (1) high-grade serous ovarian carcinoma (HGSOC), (2) low-grade serous ovarian carcinoma (LGSOC), (3) mucinous carcinoma (MC), (4) endometrioid carcinoma (EC), and (5) clear-cell carcinoma (CCC), each of which is defined by a distinct immunohistochemical profile [3]. Notably, serous carcinomas represent 75–80% of EOCs, with HGSOC being the most aggressive form of EOC and accounting for the overwhelming majority. Although both LGSOC and HGSOC belong to the serous histotype, they arise from distinct molecular events: LGSOC derives from ovarian precursor lesions, while HGSOC’s precursor lesions emerge from the fallopian tubes [4]. HGSOC, in particular, is directly associated with serous tubal intraepithelial carcinomas (STICs) as well as TP53 mutations and secretory cell outgrowths (SCOUTs) [5].

The peritoneal cavity constitutes a closed-off compartment, containing approximately 5–20 mL of peritoneal fluid that serves as a friction reliever. The main components of the peritoneal tissue are the mesothelial monolayer, responsible for the free movement of the surrounding organs; the basal lamina, composed of collagen type IV and laminin; and the submesothelial stroma, providing support [1,6]. A typical feature of ovarian cancer is its intraperitoneal route of metastasis through ascitic fluid, which is the accumulation of intraperitoneal fluid, whereas hematogenous and lymphatic routes are less common. As this fluid circulates within the peritoneal cavity, outgrowths formed by the EOC can detach from the mucosa, travel, and attach to new sites, favoring the dissemination of cancer and further contributing to its bleak prognosis [7].

Standard treatment focuses on primary surgery and/or chemotherapy based on platinum and taxanes. Despite the initial remission of the disease, however, most patients eventually have a recurrence of the disease, leading to the discouraging 5-year survival rate of <29% [8].

### 1.2. Basics of Integrin Signaling

**Figure 1:**
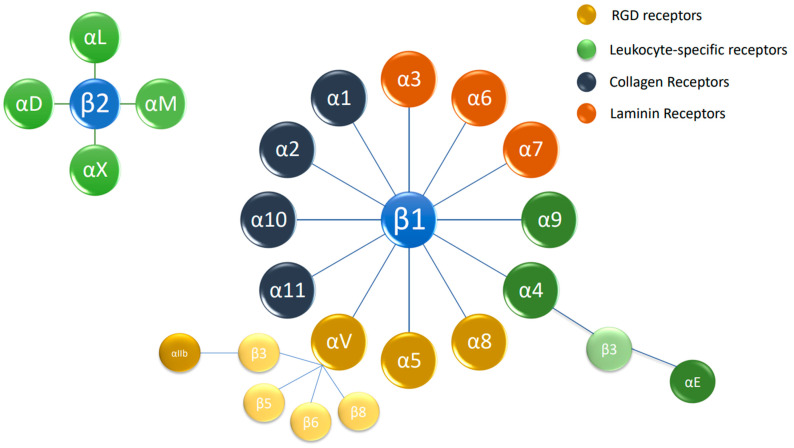
The integrin superfamily. The integrins are subdivided according to their β chains (β1 and β2 chains). Several α chains can be combined with β chains, with 24 different integrins being present in humans. RGD: arginylglycylaspartic acid.

Integrins are a family of cell-adhesion glycoprotein receptors that mediate cell–extracellular matrix (ECM) adhesion. Structure-wise, they are composed of 2 type-I protein subunits: α (18 types) and β (8 types), which heterodimerize into at least 24 unique integrins (Figure 1). The subunits consist of a transmembrane domain, an ectodomain, and a short cytoplasmic tail domain [9].

Physiologically, integrins activate bidirectional signaling pathways. While inactive, they remain connected at the short cytoplasmic tail domains of α and β subunits. Inside-out signaling, enforced by the binding of intracellular cytoskeletal proteins, such as talin, vinculin, and ERM (ezrin, radixin, and moesin), to the cytoplasmic domain of the β subunit, is of paramount importance due to its impact on the affinity of extracellular ligands [8]. As integrins are activated, they shift from a bent-closed to an extended-closed and, finally, to an extended-open conformation, which further enables the binding of extracellular ligands [10]. Outside-in signaling is implemented via the clustering of integrin receptors on the plasma membrane. Extracellular ligands, namely, numerous components of the ECM (collagen, laminin, fibronectin, and vitronectin), bind and facilitate the activation of intracellular pathways involving FAK, Rho, and Ras GTPases and adaptors (Cas/Crk and paxillin). These interactions lead to the formation of plasma membrane protrusions, called podosomes, which, in turn, allow the cells to spread [8,11].

### 1.3. Integrins in Metastasis

As mentioned above, a typical trait of EOC is its transcoelomic route of dissemination. STIC lesions, enriched with p53 mutations, form the first step of cancer development, which is enhanced by the pro-inflammatory microenvironment created by several cytokines (IL-6, IL-8) and growth factors (TGFβ, VEGF), as well as by altered cell signaling pathways [8]. An overview of this process in EOC is the initial detachment of cancer cells, followed by the formation of spheroids traveling in the ascitic fluid and, finally, attachment at secondary locations on the peritoneal mesothelium. Furthermore, a key component of the whole process is the epithelial–mesenchymal transition (EMT) [8]. At this point, the term “anoikis” should be introduced: anoikis is a special type of programmed cell death occurring in response to the loss of correct cell–ECM connections [12]. Anoikis resistance constitutes a prerequisite for cancer cells to survive their transit in the peritoneal fluid. During this whole series of molecular events, integrins, as well as other adhesion molecules, participate actively.

### 1.4. Outgrowth Formation

STIC lesions constitute a significant step toward EOC development. Before EOC cells detach, some of them take part in a process called outgrowth formation, which is characterized by the alteration of cytoskeletal dynamics, and cell–cell and cell–ECM adhesions [7]. Two models have been described: apical extrusion and apical budding.

Apical extrusion is a physiological process that takes place in normal epithelia and aims at expelling apoptotic or live cells so that tissues function properly and do not become overcrowded [13]. It is mediated by Rho-associated kinase (ROCK) non-muscle myosin II (NMMII) contractility, which facilitates cellular detachment from the basal lamina and translocation to the epithelial apex [14,15]. This translocation concerns not only healthy but also oncogene-transformed cells, which ultimately form outgrowths due to cell proliferation [16]. Laminin-binding integrin β1 might play a role in this process, enabling the re-attachment of extruded cells to the apical surface of the epithelium [7].

On the contrary, apical budding is defined by the collective colonization of cancer cells into peritoneal tissues via their apical surfaces and NNMII and ROCK activity [17]. It is suggested that this model is prevalent in STIC and CRC because of the peculiar polarity of cells (facing the peritoneal cavity). Therefore, it is possible that apically localized integrins can potentially mediate adhesion to proximal mesothelial surfaces [7].

### 1.5. Detachment and Spheroid Formation and Integrins

Regardless of the site of origin (tube and/or ovarian surface), the dissemination process requires that tumor cells become less adherent and able to detach. This step is mediated primarily by the membrane-type 1 matrix metalloproteinase (MT1-MMP or MMP-14), whose production is upregulated in EOC cells [18]. MT1-MMP exerts its effect by cleaving the α3-integrin, thus leading to looser cell adhesion [8]. Although anoikis-induced cell death impedes the survival of single detached cells, EOC cells bypass this obstacle by forming clusters of cells called spheroids. Spheroids are multicellular hypoxia-resistant aggregates that offer a great survival advantage to EOC cells, favoring their dissemination transcoelomically. Moreover, they are resistant to first-line chemotherapeutic agents for OC due to them accumulating in the G0/G1 phase of the cell cycle and are associated with faster disease progression [8,19].

Integrins are profoundly correlated with this cellular event. Gao et al. published a study in 2019 wherein the authors focused on the early onset of peritoneal dissemination in HGSOC, a characteristic that sets it apart from LGSOC. The researchers identified a type of ascitic tumor cell (ATC) in HGSOC that expresses high levels of integrin α5 (ITGA5). These cells are inclined to form heterotypic spheroids, or 3D clusters, with fibroblasts. The authors named these clusters “metastatic units” (MUs) due to their enhanced metastatic ability and their active participation in early peritoneal dissemination. Interestingly, fibroblasts within these MUs support the survival of ATCs and guide their invasion into the peritoneum. Once there, the fibroblasts become vital components of the tumor stroma in newly formed metastases. The study found that cancer-associated fibroblasts (CAFs) attract high-ITGA5 ATCs to form MUs, which further maintain the ATCs’ ITGA5 expression by secreting EGF, a growth factor. The authors noted that LGSOC, in contrast, has very few CAFs and resultant MUs, which may contribute to its slower metastatic progression. The paper concluded by suggesting that these findings present a specialized MU architecture that intensifies tumor–stroma interactions and encourages transcoelomic (across the body cavity) metastasis in HGSOC [20].

Integrins are profoundly correlated with this cellular event. The integrin β1 subunit heterodimerizes with several a-subunits and has been proven to enable EOC spheroid formation by mediating the adhesion of cancer cells to various ECM components (laminin, fibronectin, and vitronectin) [8]. Ray et al. proposed the implication of type-I leucine-rich repeat-containing membrane protein (LRRC15), adhering to fibronectin, promoting β1-clustering, and facilitating metastasis, via FAK signaling. Moreover, Ip et al. suggested that β1 integrin worked together with P-cadherin and enabled the dissemination of EOC spheroids intraperitoneally [21]. For α5β1-integrin in particular, Doberstein et al. confirmed a positive relationship between the expression of L1CAM and cluster formation. In particular, the production of fibronectin and α5β1-integrin was reported to be enhanced in EOC cell lines overexpressing L1CAM, thus promoting spheroid formation and cancer cell survival [22].

A6β1 and α2β1 represent other integrins containing the β1 subunit that are also related to spheroid formation, binding laminin and collagen, respectively. The vitronectin/αv-integrin adhesion system is also implicated in spheroid formation [23]. Carduner et al. confirmed that αv-integrin diminution in spheroids is associated with decreased ERK 1/2 activation and the induction of apoptosis [24]. Another molecule closely linked to OC is CD90 (also called Thy-1), which is a glycoprotein mostly mediating T-cell activation and neurite growth. Chen et al. established its downregulation in OC, which was associated with poor patient prognosis [25]. The authors reported that β3-integrin suppression boosted spheroid cell survival and CD133 expression and undermined the CD90-induced phosphorylation of the mTOR and AMPK signaling pathways [25]. Overall, it is suggested that β3-integrin signaling, along with CD90, exerts a negative regulatory role in anchorage-independent growth. Yin et al. found that leukocyte-specific integrins (αΜ and αX), which are abundant in tumor-associated macrophages (TAMs) and associated with β2-integrin, were able to bind with the ICAM-1 molecule, which was highly expressed in OC spheroids. Hence, the active role of TAMs in spheroid formation via integrin-mediated adhesion can be deduced [26].

### 1.6. Epithelial–Mesenchymal Transition (EMT)

The epithelial–mesenchymal transition is a morphogenetic process of cellular plasticity, where epithelial cells deviate from their epithelial phenotype (apical–basal polarity, cell–cell, and cell–ECM adhesion) and transit to a mesenchymal morphology [27]. Although this shift takes place physiologically in embryonic development, where epithelial and neuroectodermal cells acquire the capacity to migrate and drive the formation of multiple cell types, it also takes part in pathophysiological processes, such as healing, fibrosis, and cancer [23,28]. In the latter, it orchestrates some of its deadliest hallmarks (invasion, metastasis formation, and chemoresistance) [29]. Furthermore, EMT is characterized by a radical change in the expression of cell markers, namely, there is a reduction in epithelial markers (E-cadherin, β-catenin, CK8, and CK18), with an increase in mesenchymal markers, such as N-cadherin, vimentin, fibronectin, SMA, and others [30]. These effects are certainly relevant in OC as well.

Weingarten et al. demonstrated that the expression of αvβ3 integrin not only is analogous to the grade of EOC but also regulates the induction of β-catenin synthesis through the αvβ3–thyroid hormone axis and thus is vital for EMT initiation [31]. Additionally, MMP9 functions in EMT by cleaving E-cadherin, one of the aforementioned representatives of the epithelial cellular morphology, via α2β1 and α3β1 integrin signaling and upregulates fibronectin expression via α5β1 integrin signaling [8]. E-cadherin’s downregulation is also mediated by integrin-linked kinase (ILK), which binds to the β-integrin cytoplasmic tail and is enhanced via PI3/Akt signaling [30,32]. Other integrins participating in EMT are α6β4, whose expression is downregulated throughout this pathophysiological process, the laminin-binding α3β1, the collagen-I-binding α2β1-α1β1, and αvβ6, which upregulates the expression of proteases that facilitate invasion [33].

### 1.7. Mesothelial Reattachment

As said above, EOC cells transit through the transcoelomic route in the form of spheroids, thus having a greater chance of surviving in the intraperitoneal microenvironment. Invading the mesothelial tissue that surrounds the peritoneal cavity and the underlying sub-mesothelial matrix is fundamental for cell migration and the metastatic spread of cancer [34]. Another site of importance, as far as EOC metastasis is concerned, is the adipocyte-rich omental tissue. Adipocytes are tightly correlated with EOC development because they create a nutrient- and growth-factor-rich niche, which is ideal for cancer cells to thrive in [35]. The assistance of integrins in this step is pivotal once again. Huang et al. revealed that α2 integrin expression in EOC cells is decisive for their cell–collagen adhesion [36]. Furthermore, α2β1 integrin expression is associated with metalloproteinase activation (MMP2 and MMP9) as well as spheroid disaggregation, hence facilitating mesothelial invasion [37]. More specifically, MMP2 cleaves fibronectin and vitronectin into smaller fragments, to which EOC cells strongly attach via α5β1 and αvβ3 integrins, respectively [38].

Nevertheless, it has been suggested that EOC cells might adhere to the submesothelial matrix rather than the mesothelial monolayer, a molecular process called mesothelial clearance. This event takes place via EOC cells exerting an integrin- and talin-dependent force on fibronectin, according to Iwanicki et al. who first discovered it [39]. They concretely verified the implication of fibronectin-binding α5β1, while αvβ3 (fibronectin receptor) and integrins containing the α2-subunit (collagen-I receptor) did not affect the process of mesothelial clearance [39].

### 1.8. MicroRNAs (miRNAs) Are Implicated in Ovarian Cancer Development and Progression

MicroRNAs (miRNAs) are small, non-coding RNA molecules that play important roles in the regulation of gene expression. They typically function by binding to the 3′ untranslated region (3′ UTR) of target messenger RNAs (mRNAs), leading to the translational repression or degradation of the target mRNAs.

The dysregulation of miRNAs has been implicated in various human diseases, including cancer, where they can act as oncogenes or tumor suppressors depending on their target genes [40]. In ovarian cancer, several miRNAs have been found to be differentially expressed between tumors and normal tissue, suggesting their involvement in tumor initiation, progression, and metastasis, while recent data suggest their use in the prognosis and diagnosis of ovarian cancer [41,42]. For instance, the upregulation of the miR-200 family members (miR-200a, miR-200b, and miR-200c) has been reported to promote the epithelial–mesenchymal transition (EMT) and invasion in ovarian cancer cells by directly targeting the E-cadherin transcriptional repressors ZEB1 and ZEB2 [43]. This process allows cancer cells to acquire a more invasive and motile phenotype, facilitating metastasis to the peritoneum and omentum, which are common sites of ovarian cancer dissemination [40,44].

Conversely, the downregulation of tumor-suppressive miRNAs, such as the let-7 family members, has been associated with increased cell proliferation, migration, and invasion in ovarian cancer [45,46]. One proposed mechanism involves the direct targeting of the oncogene HMGA2 by let-7, whose decreased expression results in the upregulation of HMGA2 and activation of downstream pathways promoting cell cycle progression, angiogenesis, and EMT [47,48].

Moreover, miRNAs have been implicated in ovarian cancer chemoresistance, a major challenge in the clinical management of this disease. The overexpression of miR-21, for example, has been shown to contribute to cisplatin resistance in ovarian cancer cells by targeting the pro-apoptotic gene PTEN, leading to the activation of the PI3K/AKT pathway and increased cell survival [46]. Similarly, the downregulation of miR-214 has been reported to promote paclitaxel resistance through the upregulation of its target gene, β-tubulin III, which is involved in microtubule dynamics and drug efflux [49,50].

Given the potential of miRNAs as diagnostic and prognostic biomarkers, several studies have investigated their expression profiles in the circulation of ovarian cancer patients. For instance, the upregulation of miR-200a, miR-200b, and miR-200c in serum exosomes has been correlated with tumor stage and poor survival, suggesting their potential utility as non-invasive biomarkers for disease progression [51,52]. Additionally, the detection of a panel of miRNAs, including miR-21, miR-29a, and miR-92a, in the serum of ovarian cancer patients has shown promising results for early diagnosis and monitoring treatment response [53,54].

Therapeutic strategies targeting dysregulated miRNAs in ovarian cancer are also being explored, with the development of miRNA mimics and inhibitors to restore or suppress miRNA function, respectively [55]. Preclinical studies have demonstrated the potential of miRNA-based therapies in ovarian cancer, such as the use of an miR-34a mimic, which induces apoptosis and inhibits cell proliferation, migration, and invasion by targeting multiple oncogenic pathways, including Notch, Wnt, and PI3K/AKT [56]. Furthermore, the combination of miRNA-based therapies with conventional chemotherapeutic agents has shown promising results in overcoming chemoresistance and improving treatment efficacy [46,50].

### 1.9. MicroRNA Expression Amendment Could Inhibit Integrin Expression and Ovarian Cancer Progression

Integrin expression and function are tightly regulated and play critical roles in various cellular processes, such as cell migration, invasion, and proliferation, while aberrant expression of miRNAs and integrins has been implicated in pathological conditions, including cancer.

Several miRNAs have been reported to regulate ovarian cancer progression by directly targeting integrin mRNAs (Table 1). In particular, miR-29c is reported to target integrin alpha-6 (ITGA6), inhibiting ovarian cancer cell adhesion, migration, and invasion [57]. On the contrary, Fu et al. studied the expression profile of miR-93 in ovarian cancer cells resistant to cisplatin. The authors reported that miR-93 targets integrin-β8 and enhances tumor growth, angiogenesis, and resistance to cisplatin [58]. MiR-23b has been shown to downregulate the expression of integrin β3 by targeting the histone methyltransferase EZH2, which represses integrin β3 transcription (an integrin known for transforming growth factor beta-induced (TGFBI) function and paclitaxel response in ovarian cancer cells) by promoting the H3K27 trimethylation of the integrin β3 promoter [59,60]. Similarly, miR-29a can suppress the expression of integrin β1 (an integrin frequently upregulated in ovarian cancer, promoting ovarian tumorigenesis and cancer progression) by targeting the DNA methyltransferase DNMT3A, which regulates integrin β1 promoter methylation [61,62]. The miR-200 family has been reported to suppress the metastasis of ovarian cancer by downregulating integrin β1 (ITGB1) [63]. For instance, miR-200c, a member of the miR-200 family, inhibits the epithelial–mesenchymal transition (EMT) and reduces the invasive potential of ovarian cancer cells, at least in part through ITGB1 downregulation [63,64]. Similarly, miR-29b targets and downregulates ITGB1, leading to the inhibition of metastasis and invasion of ovarian cancer cells [65]. Overexpression of miR-29b in ovarian cancer cells was found to suppress cell migration and invasion, indicating a potential therapeutic strategy for ovarian cancer treatment. Another miRNA, miR-506, has been shown to inhibit ovarian cancer progression by directly targeting the integrins ITGA6 and ITGB3, both of which are implicated in ovarian cancer cell migration, invasion, and survival [66]. In ovarian cancer cells, overexpression of miR-506 significantly decreased ITGA6 and ITGB3 expression and led to reduced cell migration and invasion. miR-92a regulates the expression of integrin α5, a known key player in ovarian cancer adhesion and dissemination. The levels of integrin α5 and miR-92a expression are significantly and inversely correlated in ovarian cancer cells. The forced expression of miR-92a in cancer cells markedly suppressed peritoneal dissemination in vivo, suggesting that targeting miR-92a may prove to be a novel and effective gene therapy for patients with ovarian cancer [67]. MiR-17, whose expression was negatively correlated with ITGA5 and ITGB1 expression in various EOC cell lines, decreased cell adhesion and invasion, as well as reducing peritoneal metastatic nodules in vivo by targeting ITGA5 and ITGB1 [68].

In addition to directly regulating integrin expression, miRNAs can also modulate integrin-mediated cellular processes by targeting downstream signaling molecules. For instance, loss of HOXD10 gene expression induced by the upregulation of miR-10b accelerates the migration and invasion activities of ovarian cancer cells, which leads to the upregulation of integrin β3 expression and activation of downstream signaling pathways [69,70]. Similarly, miR-126 has been shown to regulate endothelial cell migration and angiogenesis by targeting the PI3K-Akt pathway (associated with approximately 70% of ovarian cancers), which is downstream of integrin signaling [71,72]. These findings suggest that miRNAs can modulate integrin-mediated cellular processes by targeting downstream signaling molecules.

These findings suggest that miRNAs play a significant role in the regulation of integrin expression and function in ovarian cancer, potentially providing new avenues for therapeutic intervention. Targeting miRNAs that regulate integrins or downstream signaling pathways of integrins may have therapeutic potential for inhibiting ovarian cancer development. Further understanding of the specific mechanisms through which miRNAs regulate integrin expression could aid in the development of novel therapeutic strategies for ovarian cancer.

## 2. Conclusions

Ovarian cancer development and progression have been proven to be regulated by the crucial role of integrins that promote cell adhesion and migration, thereby facilitating the metastatic spread of ovarian cancer cells. Recent studies provide evidence of the regulatory influence of microRNAs on integrin expression, shedding light on a potential therapeutic target for the treatment of ovarian cancer. MicroRNAs could possibly have a multidisciplinary role in understanding, diagnosing, prognosing/preventing, and treating this deadly disease. Future research should focus on identifying additional microRNAs and their targets and exploring the potential for microRNA-based therapies in managing ovarian cancer. By gaining a deeper understanding of these intricate relationships, we may ultimately be able to develop novel therapeutic strategies that can more effectively combat ovarian cancer and improve the prognosis for patients afflicted with this aggressive malignancy.

## Figures and Tables

**Table 1:** MicroRNAs identified to negatively regulate ovarian cancer progression via downregulation of integrins.

MicroRNA	Targeted Integrin	Stage of Ovarian Cancer Affected
miR-200 family	ITGB1	Development
miR-29b	ITGA6 ITGB1	Metastasis and invasion
miR-506	ITGA6 ITGB3	Adhesion, migration, and invasion
miR-92a	ITGA5	Metastasis and invasion
miR-93	ITGB8	Adhesion, migration, and invasion
miR-183	ITGB1	Metastasis and invasion
miR-124	AVB3	Adhesion and invasion
miR-182	ITGA5	Unknown
miR-29c	ITGA6	Adhesion, migration, and invasion

ITGA: integrin α; ITGB: integrin β; AVB3: Alpha-v beta-3.
